# Relationship between daily rhythms and verbal memory in individuals with Bipolar Disorder

**DOI:** 10.1192/j.eurpsy.2025.313

**Published:** 2025-08-26

**Authors:** K. T. Svee, A. Engum, G. Morken, T. I. Hansen

**Affiliations:** 1 Division of Mental Healthcare, St. Olavs Hospital; 2 Department of Mental Health; 3 Department of Neuromedicine and Movement Science, Norwegian University of Science and Technology, Trondheim, Norway

## Abstract

**Introduction:**

Cognitive function, particularly verbal memory, is often compromised in Bipolar Disorder (BD). While studying risk factors for cognitive deficits has not identified causal factors, focusing on protective factors that support verbal memory can help tailor interventions for individuals with BD.

**Objectives:**

Investigate associations between daily rhythms and verbal memory in people with BD in full or partial remission.

**Methods:**

This is a cross-sectional study. Participants were included if their Montgomery Asberg Depression Rating Scale (MADRS) score was ≤16 and Young Mania Rating Scale (YMRS) score was ≤8. Daily rhythms were assessed by self-report using the BRIAN scale, as was chronotype. Regularity and intensity of physical activity were measured with actigraphy, with devices worn on the wrist for up to ten days. Variables of interest included mean time per day in moderate to vigorous physical activity (MVPA), intensity and timing of the most active five hours per day (M5), and total intensity per 24 hours over the assessment period. Cognitive function was assessed using a validated, self-administered, web-based test platform for Norwegian-speaking participants, which included a verbal memory test. Actigraphy data were processed using specialized software to extract relevant metrics. Correlational analysis was conducted to evaluate the relationships between daily rhythms and verbal memory.

**Results:**

A total of 87 participants were included, comprising 30 men and 57 women, aged between 18 and 64 years. Among them, 57 had bipolar disorder type 2, and 30 had bipolar disorder type 1. The analysis revealed a significant positive correlation between verbal learning and the timing of the most active five hours, with better verbal learning observed for M5 timing later in the day. There was also a moderate positive correlation between better delayed verbal recall and the amount of time spent in moderate to vigorous physical activity.

**Conclusions:**

Our findings suggest that modifiable factors, such as later timing of the most active five hours and amount of time spent in moderate to vigorous physical activity, are associated with better verbal learning and memory in individuals with bipolar disorder. These insights could inform interventions aimed at improving cognitive outcomes in this population.

**Disclosure of Interest:**

None Declared

